# The Evaluation of the Relationship between sTREM-1, VEGF-B, and VEGF Gene Expression Levels with Disease Activity of Behçet's Patients

**DOI:** 10.1155/2018/2649392

**Published:** 2018-10-21

**Authors:** Kadir Harmanci, Ozden Yildirim Akan, Timur Pirildar, Pinar Ortan, Cevval Ulman

**Affiliations:** ^1^Department of Internal Medicine, Salihli State Hospital, Manisa, Turkey; ^2^Department of Internal Medicine, University of Health Sciences, Izmir Bozyaka Training and Research Hospital, Izmir, Turkey; ^3^Department of Neurology, University of Health Sciences, Izmir Bozyaka Training and Research Hospital, Izmir, Turkey; ^4^Department of Biochemistry, Celal Bayar University, Turkey; ^5^Department of Rheumatology, Celal Bayar University, Turkey

## Abstract

**Background:**

There is no specific marker that shows the disease activity in Behçet's disease.

**Aim:**

In this study, we aimed to investigate VEGF-B and VEGF gene expressions and sTREM-1 levels in association with the activation of Behçet's disease.

**Study Design:**

Case-control study.

**Methods:**

Clinical features of patients who applied in the rheumatology clinic and were diagnosed with BD according to the international working group's criteria were investigated. 30 healthy volunteers and 30 patients in the active period according to the EBDCAF scoring were studied. VEGF-B and VEGF gene expressions and sTREM-1 levels were studied in the serum samples of the patients and the control subjects.

**Results:**

The VEGF-B expressions and sTREM-1 levels were higher in the BD than those in the healthy group, but this difference did not reach statistical significance. VEGF gene expression was statistically significant (*p* = 0.008). Behçet's disease patients with oral aphthae, genital ulcer, eye, joint, vascular, skin, and neurological involvement were analyzed separately as subgroups. We find that VEGF gene expression level of Behçet's disease patients with joint involvement (arthritis/arthralgia) and also VEGF-B and VEGF gene expression of Behçet's disease with vascular involvement (DVT/thrombophlebitis) were significantly higher (*p* = 0.035, *p* = 0.021). Each subgroup was analyzed with the control group. We determined that VEGF gene expression in all subgroups was significantly higher than that in the control group. At the same time, VEGF-B levels of patients with genital ulcer and vascular involvement (DVT/thrombophlebitis) were significantly higher than those in the control group.

**Conclusion:**

VEGF-B and VEGF gene expressions can be activity indicators for BD. In addition, this study shows that new treatment options should be explored for Behçet's disease patients with joint and vascular involvement. In the following years, new treatment methods are needed to investigate for revealing the role of the etiopathogenesis of BD and the activation and prognosis of VEGF by examining this study and providing much more participation. In our study group, the sTREM-1 levels were high but the results did not reach statistical significance. More studies are needed with larger groups in order the highlight the exact role of STREM-1 in Behçet's disease.

## 1. Introduction

Behçet's disease (BD) is a multisystemic chronic inflammatory disease characterized by a triple symptom complex with recurrent oral ulcers, genital ulcers, and uveitis accompanied by some dermatological, vascular, neurological, leucomotor, intestinal, urogenital, and cardiopulmonary symptoms that can be with remissions and exacerbations. BD is considered to be a systemic vasculitis with a nontypical histopathologic feature that can affect a wide range of diameter and localization in both the arterial and venous system, especially with a tendency to venous thrombosis [1]. The etiopathogenesis of BD has not yet been established. However, it is now accepted that the immune system is triggered by the influence of appropriate environmental factors in genetically susceptible individuals, resulting in endothelial destruction and clinical manifestations of BD [2].

The underlying exact cause of the Behçet's syndrome is unknown. As with other autoimmune diseases, the disorder may represent aberrant immune activity triggered by exposure to an infectious agent, in patients with a genetic predisposition to develop the disease. Major disease mechanisms in Behçet's syndrome include genetic factors (in association with certain human leukocyte antigens as well as some non-HLA genes), altered host bacteria response, altered populations of hematopoietic cells and cytokines, presence of immune complexes and autoantibodies, vascular endothelial activation, and hypercoagulability.

Vascular endothelial growth factor (VEGF) is the most specific mythogenic factor for vascular endothelial cells. It is a powerful cytokine modulating angiogenesis and vasculogenesis. VEGF is produced by many different cells such as macrophages, monocytes, neutrophils, and endothelium, and its formation is triggered by proinflammatory cytokines [3]. All of these are important for the pathogenesis of Behçet's disease. Hypoxia is the major regulator of expression of the VEGF gene in both in vivo and in vitro environments. Besides the hypoxia, many cytokines, hormones, and growth factors regulate VEGF gene expression leading to the release of VEGF [4]. Polymorphisms of the vascular endothelial growth factor (VEGF) gene are one of the underlying causes of Behçet's syndrome.

TREM-1 is an immunoglobulin receptor which is expressed on the surface of macrophages, monocytes and neutrophils. The soluble form of TREM-1 (sTREM-1) is released from the cells. sTREM-1 has been reported to be a useful diagnostic marker in body fluids such as serum, saliva, cerebrospinal fluid, and lymphoid fluid [5]. In a study by Bostanci et al., they demonstrated the presence of s-TREM in saliva, in periodontal diseases [6]. BD has oral manifestations, so s-TREM levels may be a biomarker of BD. As s-TREM-1, VEGF-B, and VEGF-B gene expressions may have a role in the pathogenesis of BD, we aimed to investigate the association of VEGF-B and VEGF gene expressions and sTREM-1 levels with Behçet's disease activity in this study. In the literature, there is not a study on this topic.

## 2. Patients and Methods

30 active Behçet's patients (group 1) who were diagnosed according to the International Behçet's Disease Study Group Diagnostic Criteria that were attending Celal Bayar University Medical Faculty Rheumatology Clinic and 30 healthy subjects (group 2) were taken for the study.

We recorded the frequency of oral aphthae, genital ulceration, erythema nodosum, papulopustular rash, ocular involvement, arthritis, arthralgia, thrombophlebitis, deep vein thrombosis, neurological involvement, and gastrointestinal involvement with a detailed history and physical examination of the Behçet's disease patients. All patients were subjected to a pathergy test by immersing a 12-gauge injector needle into the forearm skin. The presence of systemic involvement in Behçet's disease was assessed with a Behçet's disease current activity form (BDCAF), and the active Behçet's disease patients were taken to the study [7]. Patients with hypertension, diabetes mellitus, and malignancy were excluded.

Whole blood was collected by venipuncture in vacutainer tubes containing ethylenedinitrilotetraacetic acid (EDTA). Centrifuged serum samples were stored at −80°C until the working day.

sTREM-1, VEGF-B, and VEGF gene expression levels were studied from the patients and from the healthy volunteers.

sTREM-1 is assessed with Cusabio Biotech Co. limited kit (Wuhan, China) by using the ELISA method from the serum. sTREM-1 interassay CV values are 12.6% while the intraassay CV values are 10.7%. The minimum measurement limit of sTREM-1 was 7.81 pg/ml and the detection limit was 31.25–2000 pg/ml.

During the determination of the VEGF gene expression levels, RNA isolation was done by using High Pure RNA isolation kit (Roche, catalog no. 11828665001) from the blood samples which were taken to purple-capped EDTA tubes. To prevent DNA contamination during RNA isolation, the samples were studied in a laminar flow cabinet. cDNAs were obtained using the Transcriptor High Fidelity cDNA synthesis kit (Roche, catalog no. 05081955001) from the isolated RNA samples. They were assessed by the RealTime Ready Catalog Assay (catalog no. 05532957001) and RealTime Ready Catalog Designer Assay (catalog no. 05583055001) protocols.

PCR efficiency was accepted as 2 for this test. The random hexamer primer was used in the cDNA step. Beta actin was used as a housekeeping gene. The primer probe UPL (Universal Probe Library) used for VEGF is a probe consisting of 8–9 mer LNA (locked nucleic acid) technology which binds OH groups and increases the melting value. The primers we used were specific for VEGF mRNA and were made using the UPL probe to make the amplified region visible.

Our method is as follows: (1) a mixture of VEGF primer probe + H_2_O + polymerase (at 5x concentration) was prepared. Capillary tubes are arranged. Capillary 15-microliter mix was distributed to the tubes. (2) Mixture of actin (housekeeping gene) primer probe + H_2_O + polymerase (at 5x concentration) was prepared. Capillary tubes are arranged. Fifteen-microliter mix was distributed to the capillary tubes. The capillaries were closed by putting 5 microliters of negative control as H_2_O, 16th, and 32nd specimens, with a total volume of 20 microliters. Then, 5 microliters of cDNA were plugged into the capillaries according to their sample numbers (at 2000 rpm), for 1 minute, and were loaded onto a real-time PCR instrument. The housekeeping genes of the same specimens were studied at 17–32 while 1–16 VEGF were studied in the same run. Amplification was performed over 45 cycles. The resulting curves were viewed from the screen. The results were calculated using the ratio of Michael Pfaffl's mathematical method: ratio = (*E*_target_) *∆* CP_target_ (control − sample)/(*E*_ref_) *∆* CP_ref_ (control − sample).

VEGF/beta actin ratios were compared; 1 log change was 10 times different. It was considered meaningful if the target/ref for example no. 1 made a tenfold difference from the target target/ref ratio after 41 of the exercise. This proportional difference is considered to be a sensitive indicator of changes in physiological expression, even if the cDNA is a minimal variation. In the future, the relative rate of expression, which is indicative of mRNA differentiation rather than quantitation models over complex and time-consuming calibration curves, is considered to be an ideal and simple tool.

## 3. Statistical Analysis

Analyses of the study were evaluated in the Statistical Package for the Social Sciences Version 12.0 (SPSS 12.0) statistical program. Averages and standard deviations of all values were calculated. Chi-square, *t*-test, and Mann–Whitney test were used for statistical analysis. All the data were expressed as mean ± SD; a *p* value of <0.05 was considered statistically significant.

## 4. Results

In our study, the mean age of the patients with active Behçet's disease consisting of 30 individuals was 37.3 ± 8.75 years, while the mean age of the control group was 30.1 ± 4.69 years. There was a statistically significant difference between the two groups (*p* < 0.05). 18 (60%) of the cases with Behçet's disease were male and 12 (40%) were female while 15 (50%) of the control group and 15 (50%) were female. There was no statistically significant difference between the two groups in terms of gender distribution (*p* = 0.436).

**Figure 1:**
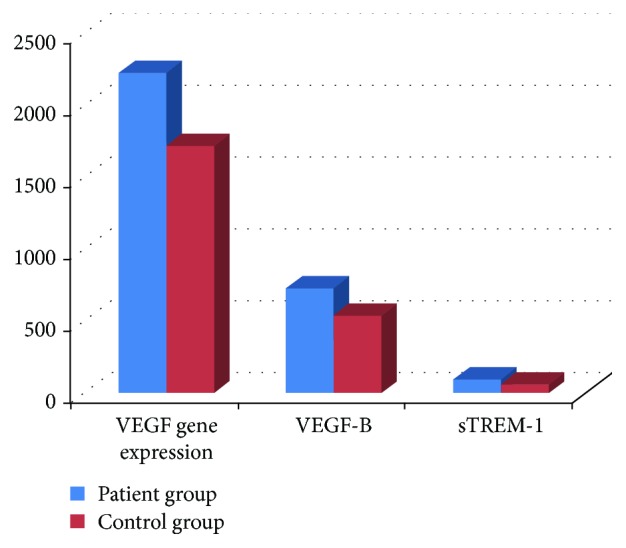
The sTREM-1, VEGF-B, and VEGF gene expression levels of Behçet's disease patients and healthy controls.

The distribution of clinical findings of patients with Behçet's disease is shown in Table 1. In the Behçet's disease group, the mean levels of VEGF gene expression were 2240.06 ± 780.62 units and 1731.14 ± 651.88 units in the control group (*p* = 0.008). The statistical comparison was significant. The mean sTREM-1 level was 735.57 ± 578.44 pg/ml while it was 549.70 ± 337.21 pg/ml in the control group. The difference was not statistically significant (*p* = 0.134. The mean VEGF-B level was 102.80 ± 122.77 pg/ml in BD and 63.81 ± 37.01 pg/ml in the control group. The difference was not statistically significant (*p* = 0.101) (Figure 1).

We analyzed the subgroups of patients as oral aphthae, genital ulcerations, eyes, joints, veins, skin, and neurologically affected subgroups separately. For each clinical indication, sTREM-1, VEGF-B, and VEGF gene expression levels of patients were compared with the sTREM-1, VEGF-B, and VEGF gene expression levels of other Behçet's disease patients. The level of VEGF gene expression was statistically significantly higher in patients with Behçet's disease with joint involvement than that with Behçet's disease without joint involvement (*p* = 0.01). In addition, VEGF-B and VEGF gene expression levels were significantly higher in Behçet's disease patients with vascular involvement (DVT/thrombophlebitis) than those without Behçet's disease (*p* = 0.035, *p* = 0.021).

No statistically significant difference was found in other clinical findings (oral aphthae, genital ulcer, ocular involvement, erythema nodosum/papulopustular rash, neurological involvement, and positivity test) (*p* > 0.05).

## 5. Discussion

BD is a chronic vasculitis involving both the arteries and the venules, with recurrent oral and genital ulcers, skin findings, and uveitis. The geographical distribution of BD on the world shows significant differences, with especially highest prevalence detected on the historic Silk Road (Turkey, Iran, Korea, Japan, etc.) [8]. There is not a specific test or laboratory method so the diagnosis is made according to the clinical findings. Therefore, the International Study Group Criteria (ISGC) established in 1990 is utilized [9].

Since there was no reliable laboratory test to reflect clinical activity in BD, the clinical findings and EBDCAF scores were used in our study to determine the active cases of BD [5]. According to these criteria, there were various combinations of clinical findings such as oral aphthae, genital ulcers, ocular involvement, erythematosus, papulopustular lesions, vascular involvement (DVT and thrombophlebitis), joint involvement (arthritis/arthralgia), and neurological involvement in the majority of Behçet's disease patients (Table 1). However, there was not any patient with an arterial aneurysm or gastrointestinal involvement.

VEGF is produced by many different cells such as macrophages, monocytes, neutrophils, and endothelium and its formation is triggered by proinflammatory cytokines [8]. As proinflammatory cytokines may play a role in the pathogenesis of BD, s-TREM levels may be elevated in the active phase of the disease. The high VEGF gene expression in active Behçet's disease patients in our study supports this association.

Erdem et al. reported that serum VEGF-A levels were significantly higher in Behçet's disease patients and that VEGF could be an activity marker in their study of 33 patients and 20 healthy control groups in order to investigate the association of Behçet's patients with VEGF-A [10].

Hamzaoui et al. measured VEGF-A levels and VEGF gene expression in cerebrospinal fluid of 32 neuro-Behçet's disease patients and compared them with the control group. In conclusion, they found VEGF-A and VEGF gene expression levels in the cerebrospinal fluid and serum in the neuro-Behçet's disease group. VEGF-A levels decreased significantly after 3 months of therapy. For this reason, Hamzaoui and colleagues have indicated that VEGF-A may play a role in the neurological involvement of Behçet's disease in the active phase [11].

While there is no study of VEGF-B in Behçet's disease patients in the literature, Mould et al. used the experimental mouse model to show the role of VEGF-B in the development of arthritis and found that the mouse was protected against synovial inflammation and the formation of joint destruction by inhibiting the binding of VEGF-B to the receptor (VEGFR-1). At the end of the study, VEGF-B has been implicated to have a major role in inflammatory angiogenesis and pathogenesis of arthritis [12].

Recent studies have shown that VEGF-B plays a role in autoimmune arthritis and tumour angiogenesis. In these studies, neovascularization has been shown to be suppressed by the blockade of VEGF-B. Again, the in vivo activity of VEGF-B was assessed using the ischemic model. In an animal model, femoral artery ligation was performed in the hind paw of the animal. Blood flow increased after 7 days of femoral artery ligation. Postmortem angiographic examination showed a significant increase in vascular density and the number of mid-to-large collateral arteries, which was associated with the expression of VEGF-B [13].

In Behçet's disease patients with joint swelling, increased intra-articular pressure disturbs tissue perfusion, while the oxygen is consumed by the metabolic activity of inflammatory cells collected in the region and their wastes accumulate due to degraded microcirculation. The body responds to this change by increasing the vascular permeability in the region when VEGF is synthesized and new vessel formation begins. In our study, the level of VEGF gene expression was statistically significantly higher in Behçet's disease patients with joint involvement. These findings suggest that VEGF gene expression may be an activation marker in Behçet's disease patients with joint involvement and that VEGF-B may suggest new approaches in the treatment of arthritis patients with Behçet's disease.

Vascular lesions that may be of a wide spectrum ranging from small vessel vasculitis to large arterial/venous lesions and superficial thrombophlebitis to deep venous thrombosis are the hallmarks of this disease. Vasculitis develops mainly as a secondary to endothelial damage and endothelial dysfunction. Studies in the literature have shown that VEGF-B increases collateral vessel development in the ischemic animal model [13]. It has been shown that mice lacking VEGF-B have a smaller heart and regeneration is impaired after experimental myocardial infarction mice model. This suggests that VEGF-B plays an important role in coronary artery regeneration and angiogenesis [14]. Silvestre et al. have shown that VEGF-B promotes angiogenesis by Akt- and eNOS-related pathways [15]. In our study, it was thought that the statistically significant levels of VEGF-B and VEGF gene expressions in Behçet's disease patients with vascular involvement (DVT/thrombophlebitis) may be due to the increased expression of VEGF-B after ischemia to stimulate new collateral development. We hope that VEGF-B and VEGF gene expressions may be activation markers in vascular BD and that new studies in this area can develop new strategies in the treatment of thrombosis.

Similarly, Çekmen and colleagues found that plasma VEGF-A levels in active Behçet's disease patients were significantly higher than those in healthy controls. They also found that VEGF levels in Behçet's disease patients with ocular involvement were significantly higher than those in the control group. This states that VEGF may play a role in dermal, ocular, and vascular events in the course of the disease [16].

TREM-1 is a new receptor from the immunoglobulin superfamily, which is expressed on the surface of monocytes, macrophages, and neutrophils. The soluble form of TREM-1 (sTREM-1) is released from the cells. sTREM-1 has been reported to be a useful diagnostic marker for body fluids [10]. In Behçet's disease, monocytes, macrophages, and neutrophils play a role in the inflammatory response. We, therefore, evaluated the association of sTREM-1 with disease activation in active Behçet's disease patients, considering that serum sTREM-1 levels may be high. Collins and colleagues found that sTREM-1 levels in the synovial fluid of patients with rheumatoid arthritis (RA) and septic arthritis were high. They noted that TREM-1 suppression in RA may result in therapeutic benefits by reducing local proinflammatory cytokine and chemokine release [17].

Park et al. compared 31 ulcerative colitis and 22 Crohn's patients with healthy controls to demonstrate the correlation between the activity of inflammatory bowel disease and serum sTREM-1 levels. The level of sTREM-1 is elevated in individuals with inflammatory bowel disease and is well correlated with disease activity. This result suggests that sTREM-1 may be an activity marker [18].

Chen and colleagues investigated the association of sTREM-1 levels with the activation of the disease in 80 ankylosing spondylitis (AS) and 30 healthy control groups. sTREM-1 levels were measured in the synovial fluid of 6 patients and in the serum of the other patients and compared with the control group. The serum and synovial fluid's sTREM-1 levels were significantly higher in the control group. It is stated that sTREM-1 may be a new parameter in the early diagnosis of AS [19]. Jung et al. showed the correlation of sTREM-1 level with disease activity in the study of 88 Behçet's disease patients with intestinal involvement. They proposed that the sTREM-1 levels may be a significant serologic marker showing disease activity in Behçet's disease [20]. In our study, sTREM-1 level in active patients with Behçet's disease was not statistically significant although it was higher than that in the control group. This may be due to the low number of patients and immunosuppressive use.

In conclusion, high expression of VEGF gene expression in active Behçet's disease patients and accompanying high VEGF-B levels in patients with vasculitis (DVT/thrombophlebitis) indicate that these immunologic markers may be activity indicators. Furthermore, new treatment options should be examined in patients with Behçet's disease with joint and vascular involvement. In the following years, there is a need for new studies that investigate this work and explore the role of VEGF, its role in the etiopathogenesis, activation and prognosis of BH, and therapies for treatment with higher participation. Our work may be a basis for these studies.

## Figures and Tables

**Table 1:** The distribution of the clinical findings of patients with Behçet's disease.

Clinical findings	Number (*n*)	Percentage (%)
Oral aphthae	23	76.7
Genital ulcerations	13	43.3
Eye symptoms	16	53.3
Papulopustular eruptions and EN	18	60
Joint involvement (arthritis/arthralgia)	12	40
Vascular involvement (DVT/thrombophlebitis)	3	10
Neurological involvement	3	10
Positive pathergy test	19	63

## Data Availability

The data used to support the findings of this study are included within the article.
